# Prostaglandin receptors EP and FP are regulated by estradiol and progesterone in the uterus of ovariectomized rats

**DOI:** 10.1186/1477-7827-10-3

**Published:** 2012-01-18

**Authors:** Chellakkan S Blesson, Edgar Büttner, Britt Masironi, Lena Sahlin

**Affiliations:** 1 Division for Reproductive Endocrinology and the Paediatric Endocrinology Unit, Department of Women's and Children's Health, Karolinska Institutet, Stockholm, Sweden

**Keywords:** Prostaglandin Receptors, Estradiol, Progesterone, ER agonists

## Abstract

**Background:**

Prostaglandins are important for female reproduction. Prostaglandin-E2 acts via four different receptor subtypes, EP1, EP2, EP3 and EP4 whereas prostaglandin-F2alpha acts through FP. The functions of prostaglandins depend on the expression of their receptors in different uterine cell types. Our aim was to investigate the expression of EPs and FP in rat uterus and to identify the regulation by estradiol, progesterone and estrogen receptor (ER) selective agonists.

**Methods:**

We performed four different rat experiments involving treatments with estradiol, progesterone and ER agonists. Real-time PCR and immunohistochemistry were employed to evaluate receptor expression.

**Results:**

Our results showed that all mRNAs and proteins of EPs and FP are expressed in the rat uterus. The expression pattern and intensity of immunostaining vary between different cell types and treatments. The mRNA expression of all EPs and FP are downregulated by estradiol and the ERalpha specific agonist PPT, whereas the ERbeta specific agonist DPN downregulates only EP2 and EP4. The protein expression however, showed an increase in EP2 and EP3 after estradiol treatment. When treated with estradiol and progesterone in combination, the expressions of EP1 and EP3 are upregulated.

**Conclusions:**

Regulation of EPs and FP expression by estradiol appears to be mainly modulated via ERalpha for EP1, EP3 and FP, while EP2 and EP4 also are affected by the ERbeta selective ligand. Our immunohistochemical data shows a cell specific regulation of prostaglandin receptors under the influence of ovarian steroids, where EP2 is estrogen regulated in all uterine tissues examined. EP1 and EP3 are upregulated by the combination of estradiol and progesterone. Thus, our observations indicate that estradiol and progesterone regulate the mRNA and protein expression of EPs and FP in a receptor and tissue specific way.

## Background

Prostaglandins are inflammatory mediators that play an important role in female reproduction [1-5]. Prostaglandin (PG) receptors are heptahelical transmembrane G protein coupled receptors and are expressed in cytoplasmic membranes [6,7]. PGE2 transduces its signal through four different receptor subtypes, EP1, EP2, EP3 and EP4 whereas the action of PGF2α is mediated by FP [8,9]. These receptors have a distinct differential affinity to ligands, biochemical properties and tissue localization [8]. PGE2 and PGF2α are key factors in female reproduction with vital functions in blastocyst spacing, implantation, decidualization and uterine contraction [2,9]. EP1, EP3 and FP cause smooth muscle contraction, whereas EP2 and EP4 contribute to the relaxation of smooth muscles [8,10]. EP2, EP3 and EP4 might play a role in the regulation of stromal edema, endometrial blood flow and blood vessel permeability [1].

Key reproductive events are under the strict control of estrogen and progesterone (P4). There are reports suggesting that sex steroids can modulate the expression of PG receptors. In mice, EPs and FP are expressed in a temporal and cell-specific manner around the time of embryo implantation and decidualization [2,3,11,12]. Further, P4 upregulates EP2 expression and estradiol (E2) augments this process; however, E2 alone downregulates EP2 [3]. In rats, EP2 mRNA levels are upregulated during pregnancy and their levels decline during labor and postpartum, suggesting a possible involvement of P4 in the regulation of EP2. Moreover, P4 upregulates EP2 mRNA in the uterus of ovariectomized rats [13]. A recent study on human endometrium showed that the expression of PG receptors varies throughout the menstrual cycle in a phase specific manner with EP2, EP3 and EP4 dominating the mid-secretory phase, EP1 dominating early-secretory phase and FP peaking during the proliferative phase [1] indicating that E2 and P4 might regulate their expression. Thus there are both direct and indirect results indicating steroidal regulation of PG receptors in different species. These results add to the existing complexity of the regulation of the reproductive cycle suggesting interplay between the sex steroid hormone and PG systems.

We hypothesized that E2 and P4 might regulate the spatio-temporal expression of PG receptors in the rat uterus. E2 acts via its receptors, estrogen receptor (ER) α and β. It is not known if both these receptors are involved in the regulation of PG receptors. Further, it is also not known if there is any dose or time dependent regulation of PG receptors by E2 alone and in combination with P4. The aim of this study is to evaluate cell specific expression of PG receptors EPs and FP in the rat uterus and how they are regulated by E2, P4 and ER selective agonists.

## Methods

### Reagents

Estradiol-17β and progesterone were purchased from Sigma Chemical Co. St. Louis, MO, USA. The hormones were dissolved in 99.5% ethanol at a high concentration and then diluted with propylene glycol to the required concentration. The final concentration of ethanol in the injections was less than 5%. The ERα selective ligand 4,4',4''-(4-Propyl-[1H]-pyrazole-1,3,5-triyl)trisphenol (PPT) and the ERβ selective ligand 2,3-bis(4-Hydroxyphenyl)-propionitrile (DPN) were purchased from Tocris Bioscience, Bristol, UK. RNA*later*^® ^was purchased from Ambion, Austin, TX, USA.

### Animals

Adult female rats (55-60 days old, approx. 250 g) of the Sprague-Dawley strain (BK-Universal, Sollentuna, Sweden) were used in all four experiments. The animals were housed in a controlled environment at 20°C on an illumination schedule of 12L: 12D. The rats had unlimited access to standard pellet food and water. Rats were ovariectomized and given two weeks to rest before proceeding with the treatments. The studies were approved by the committee on animal care in Sweden.

### Study 1, Effect of estradiol dose

The ovariectomized rats (n = 22) were divided into four groups. Three groups were treated with E2 subcutaneously at doses 1.0 μg (n = 4), 2.5 μg (n = 6) or 5 μg (n = 6) per rat and day for two days. The control group (n = 6) was treated with vehicle alone.

### Study 2, Effect of time

The ovariectomized rats (n = 27) were divided into four groups and treated subcutaneously with 2.5 μg of E2 daily for 1 day (n = 7), 4 days (n = 7) or 7 days (n = 6). The control group (n = 7) received vehicle alone.

### Study 3, Effect of estradiol and progesterone

Forty-two ovariectomized rats were divided into seven groups (n = 6). They were treated by subcutaneous injections with either vehicle, 2.5 μg of E2 per rat and day, and/or 1 mg P4 per rat and day. They were treated with E2 and/or P4 for 24 or 48 hours. The treatment sequence is shown in Table 1.

**Table 1:** Treatment schedule of study 3

Groups	Day 1	Day 2	Day 3
*OvxC*	vehicle	vehicle	Sacrificed
*24E*	E2	Sacrificed	
*24P*	P4	Sacrificed	
*48EE*	E2	E2	Sacrificed
*48PP*	P4	P4	Sacrificed
*48EP*	E2	P4	Sacrificed
*48PE*	P4	E2	Sacrificed

OvxC (Ovariectomized controls), E2 (Estradiol 17-β, 2.5 μg/rat), P4 (Progesterone, 1 mg/rat)

### Study 4, Mode of action of estradiol

Four groups of ovariectomized rats (n = 31) were treated with vehicle, E2, ERα selective or ERβ selective agonists, PPT or DPN, respectively. The animals were subcutaneously administered either 5.0 μg of E2 (n = 8) or 1.25 mg of PPT (n = 8) or 3.125 mg of DPN (n = 7) or vehicle (n = 8). The rats were killed 24 h after treatment. The doses of PPT and DPN were chosen by comparing data on the activity of the agonists and were prepared as reported earlier [14].

### Tissue Collection

At sacrifice, the rat uterus was removed, stripped of fat and connective tissue. In study 1 and 4 one portion was stored in RNA*later*^® ^for RNA preparation and another portion was immersion fixed in buffered formalin at room temperature (RT) overnight and then stored in 70% ethanol in 4°C to be embedded for immunohistochemistry. In study 2 and 3, tissues were only prepared for immunohistochemistry.

### RNA preparation and reverse transcription

Total RNA from uterine tissues were purified with RNeasy^® ^Mini kit (Qiagen GmbH, Hilden, Germany) according to the manufacturer's protocol for fibrous tissues. The protocol included an on-column DNase digestion, as recommended by the manufacturer. Two micrograms of total RNA from each sample was reverse transcribed at 37°C for 60 min in a final volume of 20 μl with a reaction mixture (Qiagen) containing 1 × RT buffer, dNTP mix (0.5 mM each dNTP), 600 ng random primers (Invitrogen, Paisley, UK), 30 units RNase inhibitor (Qiagen GmbH, Hilden, Germany), and 4 U of Omniscript™ reverse transcriptase (Qiagen).

### Real time PCR analysis

The oligonucleotide primers for EP1, EP2, EP3, EP4, FP and Rplp0 (housekeeping gene) are presented in Table 2, along with their predicted sizes. Real time PCR was performed in an iCycler™ iQ Real Time PCR System (Bio-Rad Laboratories, Inc, CA USA). For PCR, the cDNAs corresponding to 50 ng RNA were added to 12.5 μl of iQ™ SYBR^® ^Green Supermix (Bio-Rad) and 0.3 μM of each oligonucleotide primer in a final volume of 25 μl. After initial incubation for 3 min at 95°C, the samples were subjected to 40 cycles of 10 s at 95°C, followed by 45 seconds annealing at 57 to 64°C depending upon the genes (Table 2). All PCR assays were performed in duplicates. The purity of PCR products was confirmed by a melting curve analysis in all experiments. Each PCR assay included a negative control containing an RNA sample without reverse transcription. The primer pairs (Table 2) were designed with NCBI/Primer-BLAST program.

**Table 2:** Oligonucleotide primers used for real-time PCR

Gene	**Accession No**.	Primers F = forward; R = reverse	Product Size	**Annealing Temp**.
*EP1*	NM_013100.1	F: 5'-AGCCCCCTGCTGGTATTGGT-3' R: 5'-AGGATCTGGTTCCACGACGCGA-3'	107	63
*EP2*	NM_031088.1	F: 5'-GCTCCCTGCCTTTCACAATCTTTGC-3' R: 5'-TGGAGCTTCCGGTGCTCTCAGT-3'	200	60
*EP3*	NM_012704.1	F: 5'-ACTGTCCGTCTGCTGGTCGC-3' R: 5'-TGGTTCAGCGAAGCCAGGCG-3'	147	64
*EP4*	NM_032076.3	F: 5'-ATTCCGCTCGTGGTGCGAGTGT-3' R: 5'-CACCAATGCGGCAGAAGAGGCA-3'	202	60
*FP*	NM_013115.1	F: 5'-TCAGCAGCACAGGCAAGGCA-3' R: 5'-TGGCCATCGTCACCAGAAAGGGA-3'	110	64
*Rplp0*	NM_001002.3	F: 5'-GGCGACCTGGAAGTCCAACT-3' R: 5'-CCATCAGCACCACAGCCTTC-3'	149	57

### Immunohistochemistry

Paraffin embedded blocks were cut in 5 μm thick sections and mounted on histological slides. The paraffin sections were deparaffinized, rehydrated and washed with phosphate-buffered saline (PBS; pH 7.4). Antigen retrieval was performed by boiling the sections in 0.01 M sodium citrate buffer (pH 6.0) in microwave for 10 min. The endogenous peroxidase activity was quenched by placing the slides in 3% hydrogen peroxide in methanol. The slides were then blocked with 1.5% normal donkey serum followed by incubation with primary antibodies. Primary antibodies (Cayman Chemicals, Michigan, USA) were incubated overnight at 4°C. The antibodies were incubated by diluting to final concentrations of 3 μg/ml for EP1 (Cat # 101740), 2 μg/ml for EP2 (Cat # 101750), 1.5 μg/ml for EP3 (Cat # 101760), 10 μg/ml for EP4 (Cat # 101775) and 5 μg/ml for FP (Cat # 101802). The specificities of the antibodies were verified by performing western blot using rat uterine extracts (data not shown). These antibodies have been used and verified before in rat tissues [15,16]. The negative controls were obtained by replacing the primary antibody with an equal amount of rabbit IgG. Thereafter, incubation with biotin conjugated donkey anti-rabbit secondary antibody (SantaCruz, CA, USA) was performed at RT for 30 min at a dilution of 1:1000. Further, incubation with avidin-biotin horseradish peroxidase complex was performed at RT for 30 min using Vectastain elite^® ^ABC kit (Vector labs, CA USA). The location where the enzyme was bound was visualized by the addition of the substrate 3,3'-diaminobenzidine (DAKO, CA, USA), a chromogen which produces a brown insoluble precipitate. The sections were then counterstained with haematoxylin, which was followed by dehydration and mounting with Pertex. A Leica microscope was used to evaluate the slides. The staining intensities were assessed semi-quantitatively by manual scoring. All the slides were evaluated for the five different tissue compartments (luminal epithelia, glandular epithelia, stroma, myometrium and blood vessels). Scoring was performed independently by two investigators blinded to the identity of the slides. The staining intensity was arbitrarily graded on a scale of (0) no staining, (1) faint, (2) moderate and (3) strong.

### Statistics

Statistical analyses for all studies were carried out by Kruskal-Wallis test (ANOVA on ranks) and the significances were evaluated by Dunn's test using Sigma plot^® ^software. Values are considered significant when *P *< 0.05.

## Results

### Study 1, Regulation of EP and FP receptors by different doses of estradiol

**Figure 1:**
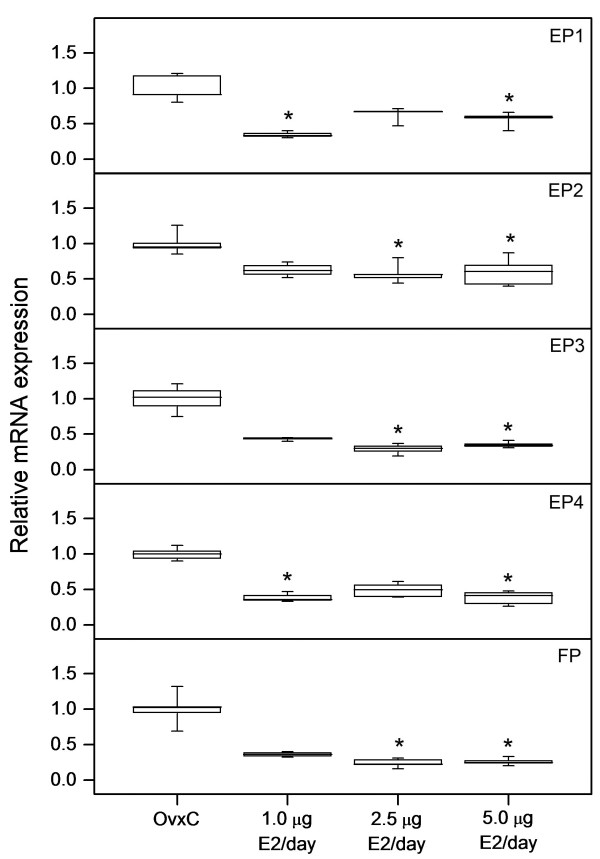
**Regulation of EP and FP mRNA by estradiol**. The relative mRNA expression levels of EP1, EP2, EP3, EP4 and FP as measured by real-time PCR in uteri from ovariectomized rats treated with different doses of estradiol (E2) along with vehicle treated controls (OvxC). Box and whisker plots representing the median value with 50% of all data falling within the box. The whiskers extend to the 5th and 95th percentiles. Bars with asterisks are significantly (*P *< 0.05) different from OvxC.

**Figure 2:**
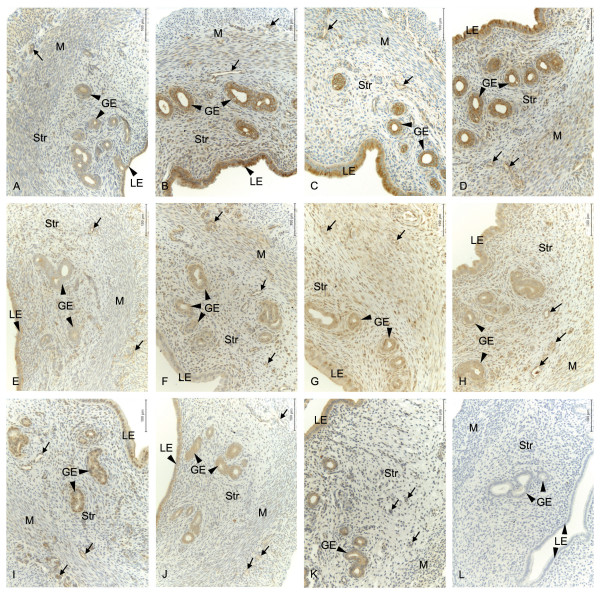
**Localization of EP and FP protein and their regulation by estradiol**. Immunohistochemical localization of prostaglandin receptors in luminal epithelium (LE), glandular epithelium (GE), stroma (Str), myometrium (M) and blood vessels (arrows) in the uteri of ovariectomized controls and different doses of estradiol (E2) treated rats. Representative images of EP2 (A-D); OvxC (A), treatment with 1 μg E2 (B), 2.5 μg E2 (C) and 5 μg E2 (D), EP3 (E-H); OvxC (E), treatment with 1 μg E2 (F), 2.5 μg E2 (G) and 5 μg E2 (H). Representative images of EP1 (I), EP4 (J), FP (K) immunostaining and a negative control (L). Magnification - 200X, Scale bar = 100 μm.

**Figure 3:**
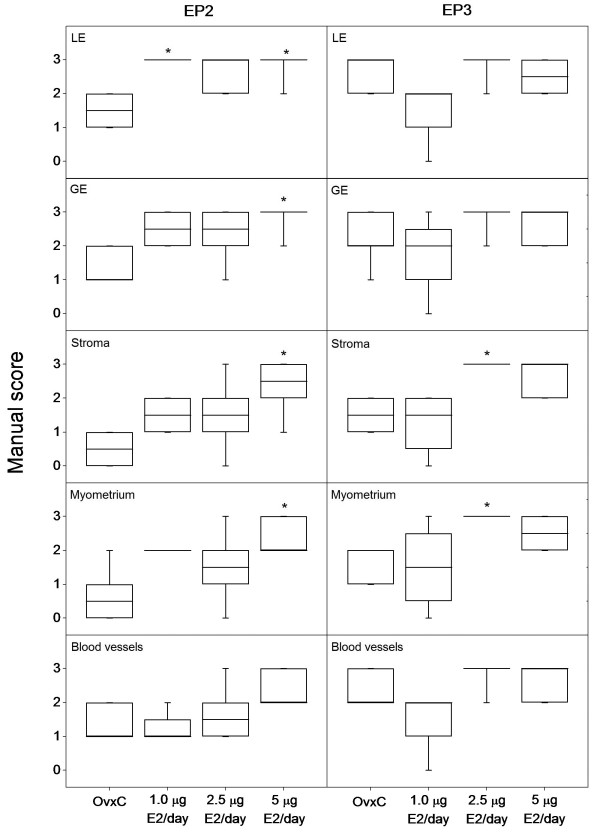
**Regulation of EP2 and EP3 protein by different doses of estradiol**. Results from manual scoring of EP2 and EP3 immunostaining in different uterine cell types when treated with different doses of estradiol (E2). Box and whisker plots representing the median value with 50% of all data falling within the box. The whiskers extend to the 5th and 95th percentiles. Bars with asterisks are significantly (*P *< 0.05) different from the control group (OvxC). LE- luminal epithelium, GE- glandular epithelium.

Real-time PCR data showed that E2 downregulates the expression of EPs and FP mRNAs in the uterus (Figure 1). The decrease was significant (p < 0.05) for EP1 and EP4 at low dose treatment (1.0 μg); EP2, EP3 and FP at mid dose (2.5 μg) and for the expression of all receptors when treated with the highest dose (5.0 μg). However, results from immunohistochemistry showed that there is an increase in the immunostaining of EP2 and EP3 following E2 treatments (Figure 2 and 3). There is an increase (p < 0.05) in the immunostaining of EP2 when treated with 5.0 μg of E2 in luminal epithelium, glandular epithelium, stroma and myometrium when compared to controls. In blood vessels a similar tendency was seen. EP3 immunostaining was increased (p < 0.05) in stroma and myometrium when treated with 2.5 μg of E2 (Figure 3). Corresponding tendencies could be observed in stroma and myometrium of study 3 after a comparable E2 treatment. Luminal epithelium, glandular epithelium and blood vessels also show similar trends. Immunostaining of the EP1, EP4 and FP receptors did not show any significant differences (data not shown).

### Study 2, Time dependent upregulation of EP2 receptor proteins by estradiol

**Figure 4:**
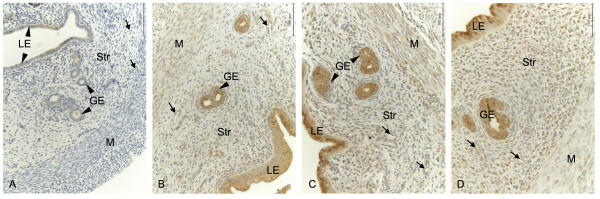
**Temporal regulation of EP2 protein by estradiol**. Immunohistochemical localization of prostaglandin receptors in luminal epithelium (LE), glandular epithelium (GE), stroma (Str), myometrium (M) and blood vessels (arrows) in the uterus of ovariectomized rats. Representative images of EP2 (A-D); OvxC (A), treatment with estradiol (E2) for 1 day (B), 4 days (C) and 7 days (D). Magnification - 200X, Scale bar = 100 μm.

**Figure 5:**
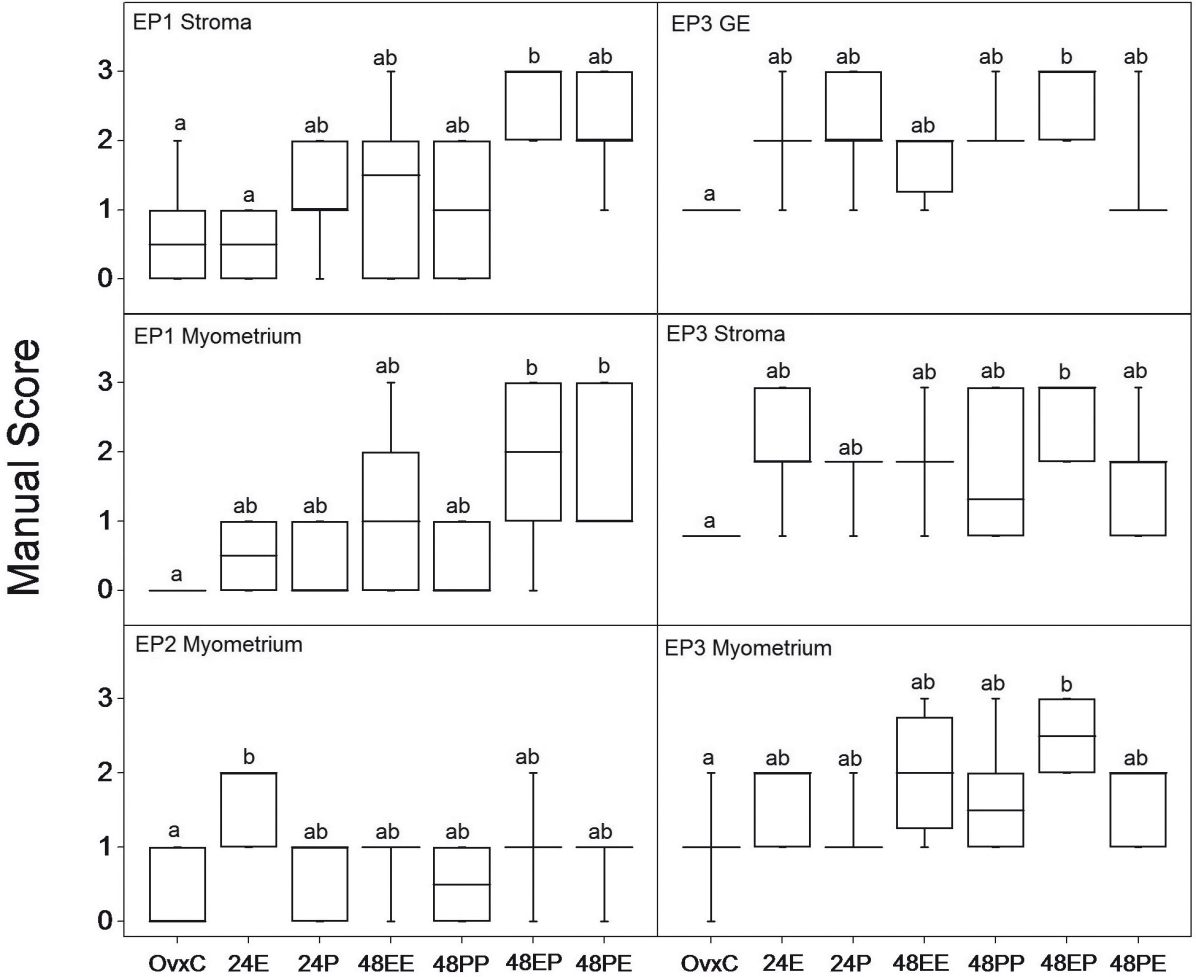
**Cell type specific regulation of EP1, EP2 and EP3 by estradiol and progesterone**. Results from manual scoring of EP1 (stroma and myometrium), EP2 (myometrium) and EP3 (glandular epithelium (GE), stroma and myometrium) when treated with estradiol (E), progesterone (P), or combinations for 24 (E or P) and 48 hours (EE, PP, EP or PE). Box and whisker plots representing the median value with 50% of all data falling within the box. The whiskers extend to the 5th and 95th percentiles. Values with different letter designations are significantly different (*P *< 0.05).

Immunohistochemical scores showed a time depended upregulation of EP2 in rat uteri when treated with 2.5 μg of E2 (Study 2). An increase (p < 0.05) in immunostaining could be observed in myometrium from day 1 but in luminal epithelium, glandular epithelium and stroma the increase was significant after day 4 (Figure 4). The increase (p < 0.05) was significant in all the cell types including the blood vessels after 7 days of treatment with E2. Our observations from studies 1 and 3 show that EP2 is not upregulated after treatment with 2.5 μg of E2 for 2 days (Figure 3) or 1 day respectively, except for myometrium on day 1 (Figure 5). The myometrial EP2 expression showed similar upregulation after day 1 in both study 2 (Figure 4) and study 3 (Figure 5) where a similar dose of E2 was used. The other PG receptors examined did not show significant regulation after E2 treatment (data not shown).

### Study 3, Regulation of EP and FP receptors by combined estradiol and progesterone treatment

In general, it was observed that there is a strong tendency for increased immunostaining of EP1 in all the compartments when treated together with E2 and P4, irrespective of the sequence of the treatments (Figure 5). In stroma we could observe an increase in immunostaining (p < 0.05) when treated with E2 followed by P4, when compared to vehicle treated control or E2 alone. Further, in myometrium an increase in immunostaining (p < 0.05) could be observed between the controls and groups treated with E2 and P4 irrespective of the treatment sequence. EP3 expression was upregulated (p < 0.05) when treated with P4 after priming with E2 in glandular epithelium, stroma and myometrium (Figure 5). Luminal epithelium and blood vessels showed similar tendencies although not statistically significant (data not shown). EP2 was upregulated (p < 0.05) in myometrium when treated with E2 alone after 24 h (Figure 5) but there was no change in the other cell types in any of the combined treatments. Treatment with P4 alone did not affect the regulation of any of the PG receptors investigated in this study. EP4 and FP did not show any regulation when treated with E2 or P4 alone or in combination.

### Study 4, Estradiol and ER selective agonists regulate the expression of EPs and FP receptor

**Figure 6:**
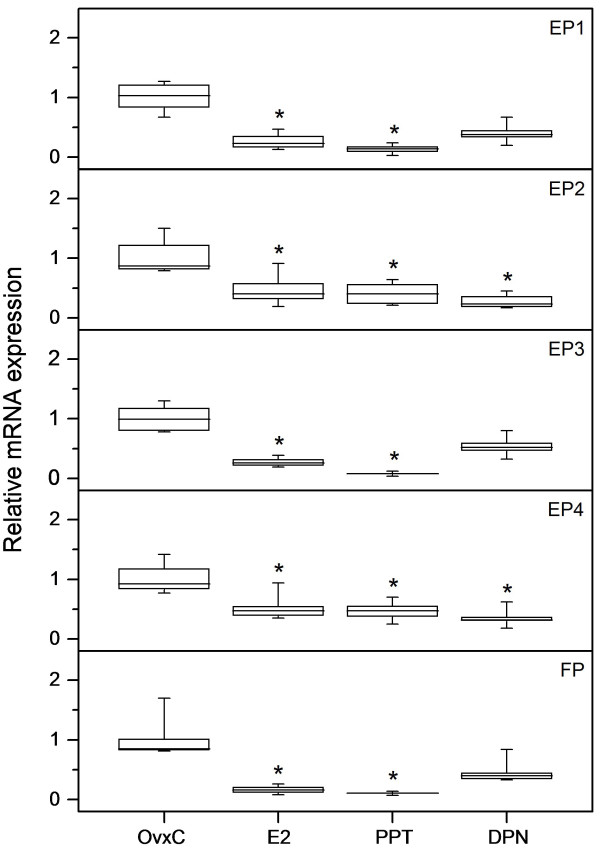
**Downregulation of EP and FP mRNA by estradiol, PPT and DPN**. The relative mRNA expression levels of EP1, EP2, EP3, EP4 and FP as measured by real-time PCR in uteri from ovariectomized rats treated with estradiol (E2), PPT or DPN along with vehicle treated controls (OvxC). Box and whisker plots representing the median value with 50% of all data falling within the box. The whiskers extend to the 5th and 95th percentiles. Bars with asterisks are significantly (*P *< 0.05) different from OvxC.

Our results from real-time PCR analyses show that 5.0 μg of E2 and the ERα agonist PPT downregulated (p < 0.05) mRNA expression for all EPs and FP (Figure 6). Treatment with the ERβ agonist DPN resulted in a downregulation (p < 0.05) of EP2 and EP4 mRNA expressions (Figure 6). However, immunohistochemical scores showed an upregulation (p < 0.05) of the EP2 and EP4 proteins in stroma when treated with E2 (data not shown).

A summary of immunohistochemical results of the significantly regulated receptors from all the four studies are given in table 3.

**Table 3:** Summary table of the significant immunohistochemistry results showing the regulatory effect

Receptor	LE	GE	Stroma	Myometrium	Blood Vessels
***EP1***	**-**	**-**	Increased (48EP)	Increased (48EP and 48PE)	**-**
***EP2***	Increased (1 and 5 μg E2 48 h, 2.5 μg E2 4 and 7 days)	Increased (5 μg E2 48 h, 2.5 μg E2 4 and 7 days)	Increased (5 μg E2 24 h and 48 h, 2.5 μg E2 4 and 7 days)	Increased (5 μg E2 48 h, 2.5 μg E2 1, 4 and 7 days )	Increased (2.5 μg E2, 7 days)
***EP3***	**-**	Increased (48EP)	Increased (2.5 μg E2 48 hrs; 48EP)	Increased (2.5 μg E2 48 hrs; 48EP)	**-**
***EP4***	**-**	**-**	Increased (5 μg E2 24 h)		**-**

48EP = 2.5 μg estradiol for 24 h followed by 1 mg progesterone for 24 h; 48PE = 1 mg progesterone for 24 h followed by 2.5 μg estradiol for 24 h; E2- Estradiol 17-β

## Discussion

Our study shows that EPs and FP are localized in different cell types of the rat uterus suggesting their involvement in normal uterine physiology. Although PG receptors are G protein coupled receptors, they are also localized to cytoplasm and nucleus [5,7,10,17,18]. We observed EPs and FP in cytoplasm and membranes, although some nuclear staining was seen in EP1 and EP4. Nuclear PG receptors have been reported to perform intracrine signaling by activating transcription [18,19].

The mRNA data shows that EPs and FPs are regulated by E2. It has been reported that P4 does not regulate mRNA of EPs, but upregulated EP2, EP3 and EP4 proteins in rat cervix [20]. In the uterus of ovariectomized rodents EP2 mRNA increased when given P4 alone or co-treated with E2 [3,13]. We did not have mRNA data to compare, but upregulation of EP2 protein levels by P4 was not found in our study. EP2 mRNA expression was downregulated by E2 in mice [3] which is in agreement with our observation in rats. Previous studies have shown the regulation of EP2, EP3 and EP4 mRNAs by E2 in pseudopregnant endometrium [21] and EP2, EP3 and EP4 protein by P4 in rat cervix [20] showing steroidal regulation of EPs. However, most conclusions in previous studies on mice are based only on RNA levels [2,3,11,13,21] and few studies shows the regulation of protein levels [5,12]. We showed that the ERα selective agonist PPT downregulates all EPs and FP mRNAs, whereas the ERβ agonist DPN downregulates EP2 and EP4 mRNAs. We did not observe any differences in protein expression after PPT or DPN treatment. This could be due to the short duration of the treatments. These observations indicate that ERα may play a major role in the regulation of EPs and FP mRNA expression. It is known that ERα is the dominant ER subtype in rat uterus [22]. The expression of EP2 and EP4 are decreased also by DPN in addition to E2 and PPT. Thus, ERβ could be involved in relaxation of the uterine smooth muscle cells. The mRNA level of FP was decreased by E2 and PPT, but not DPN treatment, indicating an ERα mediated FP regulation. None of the PG receptors inducing contraction are affected by DPN.

Our study shows the expression of EPs and FP proteins are differentially regulated by ovarian steroids. Interestingly, EP2 and EP4 proteins are exclusively regulated by E2 but EP1 and EP3 proteins are regulated only by a combination of E2 and P4. FP protein levels appear to be insensitive to ovarian steroids. Thus, the contraction mediating receptors are regulated exclusively by a combination of E2 and P4, while the relaxatory receptors are regulated only by E2. EP2 regulates epithelial differentiation and implantation [3,12,23]. E2 is known to induce proliferation and changes in epithelium in preparation for implantation [24]. Thus, regulation of EP2 by E2 in epithelium may be important for implantation. Further, EP2, EP3 and EP4 are known to play vital roles in decidualization [21]. Our findings show that administration of P4 after E2 priming upregulates EP1 and EP3, indicating the role of both steroids in the regulation of EPs in stroma during decidualization. These receptors may be regulated via a P4 mediated pathway [20]. EP4, upregulated in stroma by E2 in our study, is known to trigger decidualization under the direction of ovarian steroids [21]. PG receptors can constrict (EP1 and EP3) or dilate (EP2) blood vessels [23,25,26]. Treatment with E2 increased EP2 protein in blood vessels indicating that uterine blood flow could be regulated by E2 via EP2. It was recently reported that the expression of EP2, EP3 and EP4 increased in human endometrium during the mid-secretory phase, coinciding with increased stromal edema, blood vessel permeability and blood flow [1]. We could also show that E2 upregulates the expression of EP2 and EP3 proteins in myometrium. Interestingly, when treated with E2 in combination with P4, there is an upregulation of EP1 and EP3 also in myometrium. It appears that E2 and P4 act synergistically to increase the expression of EP1 and EP3 and estrogen priming seems to be especially important for EP3. Thus, EPs may modulate the contractile and relaxatory functions under the regulation of steroid hormones.

E2 downregulates the mRNA levels of EPs and FP, but their protein levels were either upregulated or unchanged suggesting a multilayered regulation of RNA and protein. This apparent discrepancy has to be interpreted with caution. It is not possible to compare mRNA results with protein, as RNA data shows the total expression while immunohistochemistry detects the protein in a cross section. However, it is interesting to note that such discrepancies have been reported before [1,12,20,27]. Although there are justifications based on the techniques used, we speculate that there may be a post-transcriptional regulation. Estrogen is known to regulate mRNA stability of many genes [28], and could be the possible regulator. A weakness of the present study is that only RNA data from studies 1 and 4 are analyzed. It would have added strength to the study if RNA levels from studies 2 and 3 had been available, enabling studies of long duration of E2 treatment and E2 and P4 combined effects.

The exact mechanisms of the steroidal regulation of PG receptors at transcription and translation are not clear. There are reports showing the presence of response elements in PG receptor genes that could be utilized by E2 and P4 to regulate their expression. EP1 has an estrogen response element and AP1 element [29,30], EP2 has a progesterone receptor binding element [31], EP3 and EP4 have SP1 binding sites and the FP promoter contains AP1 and SP1 sites [32,33]. Thus, it is possible that E2 and P4 directly or indirectly regulate the expression of PG receptors through these binding sites. E2 can also bind to G-protein coupled estrogen receptor-1 (GPER) to activate kinase mediated pathways [34] which may also regulate the expression of PG receptors.

## Conclusions

Our study shows that EPs and FP mRNAs and proteins are regulated by E2 and P4. E2 appear to modulate the regulation of EP1, EP3 and FP via ERα, while EP2 and EP4 could also be regulated via ERβ. Immunohistochemical data shows a cell specific regulation of PG receptors under the influence of ovarian steroids. EP2 is estrogen regulated in all uterine cell types examined whereas EP1 and EP3 are upregulated by the combination of E2 and P4 in stroma and myometrium. Further investigations are required to dissect the exact molecular mechanism by which steroid hormones regulate the expressions of PG receptors.

## Competing interests

The authors declare that they have no competing interests.

## Authors' contributions

CSB and EB performed immunohistochemistry. CSB, EB and LS scored the immunostaining. Analysis of mRNA was carried out by BM. CSB and BM performed statistical analysis of the data. CSB and LS conceived the study and drafted the manuscript. All authors read and approved the final manuscript.
